# Non-Invasive Assessment of the Seasonal Stress Response to Veterinary Procedures and Transportation of Zoo-Housed Lesser Anteater (*Tamandua tetradactyla*)

**DOI:** 10.3390/ani12010075

**Published:** 2021-12-30

**Authors:** Gabina V. Eguizábal, Mariella Superina, Rupert Palme, Camila J. Asencio, Daniel P. Villarreal, Luciana Borrelli, Juan M. Busso

**Affiliations:** 1 Instituto de Investigaciones Biológicas y Tecnológicas (IIBYT), Facultad de Ciencias Exactas, Físicas y Naturales (FCEFyN), Centro Científico Tecnológico (CCT) Córdoba-Consejo Nacional de Investigaciones Científicas y Técnicas (CONICET), Universidad Nacional de Córdoba (UNC), Av. Vélez Sarsfield 1611, Córdoba X5016GCA, Argentina; camila.asencio@mi.unc.edu.ar; 2 Instituto de Ciencia y Tecnología de los Alimentos, FCEFyN-UNC, Av. Vélez Sarsfield 1611, Córdoba X5016GCA, Argentina; 3 Laboratorio de Técnicas No Invasivas, CONICET-Parque de la Biodiversidad (ex Jardín Zoológico Córdoba), Rondeau 798, Córdoba X5000AVP, Argentina; 4 Laboratorio de Medicina y Endocrinología de la Fauna Silvestre, Instituto de Medicina y Biología Experimental (IMBECU), CCT-CONICET Mendoza-Universidad Nacional de Cuyo, Av. Ruiz Leal s/n, Parque Gral. San Martín, Mendoza 5500, Argentina; msuperina@mendoza-conicet.gov.ar; 5 Department of Biomedical Sciences, University of Veterinary Medicine, Veterinärplatz 1, 1210 Vienna, Austria; rupert.palme@vetmeduni.ac.at; 6 Parque de la Biodiversidad (ex Jardín Zoológico Córdoba), Rondeau 798, Córdoba X5000AVP, Argentina; danielvillarreal1@hotmail.com; 7 Estación de Fauna Autóctona, Secretaria de Ambiente y Desarrollo Sustentable, Santiago de Estero 2245, Salta C1067ABB, Argentina; luchiborrelli@hotmail.com; 8 Facultad de Ciencias Agrarias y Veterinarias, Universidad Católica de Salta, Salta A4400EDD, Argentina

**Keywords:** behaviour, faecal cortisol metabolites, Pilosa, veterinary check, transport, season, wildlife, ex situ

## Abstract

**Simple Summary:**

Wild mammals under human care are routinely exposed to management procedures that could be stressful and affect their welfare. Besides, wild animals in captivity are frequently housed under natural climatic conditions, which could also be a year-round challenge that affects how they cope with human intervention. Therefore, stress responses to seasons and management procedures of zoo-housed lesser anteaters (*Tamandua tetradactyla*) were non-invasively monitored. Behavioural and physiological stress responses differed between seasons. Then, lesser anteaters were studied during winter and summer by exposing them to a routine veterinary check and transportation. Behaviour before and after the procedures was video-recorded and analysed for possible alterations of activity. Faecal glucocorticoid metabolites were measured to evaluate adrenocortical activity. Both procedures altered only behavioural activities, and the response was stronger in summer than winter. We conclude that routine procedures may only slightly compromise the welfare of zoo-housed lesser anteaters, affecting them less in winter than in summer.

**Abstract:**

Management procedures affect behavioural and physiological stress responses of wild mammals under human care. According to the Reactive Scope Model, normal values are presumed to exist within predictive and reactive ranges. First, stress parameters of zoo-housed adult *Tamandua tetradactyla* were evaluated in winter and summer (29 days each), determining the level of behaviour and/or physiological parameters needed to respond to predictable environmental changes. Secondly, the effects of veterinary procedures and transportation were studied in both seasons. Non-invasive methods were applied, assessing behaviour through videos and adrenocortical activity by faecal glucocorticoid metabolites (FGMs). Lesser anteaters exhibited seasonality (summer > winter) in some behavioural parameters, such as nocturnal activities, as well as in the activity cycle (e.g., acrophase) and FGMs. A veterinary check elicited an increase in total activity (TA), natural behaviours and repetitive locomotion and affected the activity cycle, particularly in summer. Transport produced changes in TA, nocturnal and natural activity and some variables of the activity cycle, mostly during summer. Although the effects of routine management procedures were different from each other and presumably stressful, they elicited changes only at the behavioural level, which was greater during summer. The differences observed according to non-invasive methodologies highlight the importance of a multidisciplinary approach in this context and suggest that it is unlikely that individual welfare was affected.

## 1. Introduction

Wild animals under human care are often exposed to routine management procedures (e.g., capture and transportation) that may cause different behavioural and physiological changes (stress responses), possibly affecting their physical health and welfare [1,2]. Since zoos are required to maintain high standards of animal welfare, less invasive tools are desirable to minimize intervention in wild animals when studying behavioural and physiological issues [3,4].

Over the past few years, more and more non-invasive methodologies have been employed to monitor stress responses of wild animals under human care. For instance, the use of video technology to monitor behaviour has improved opportunities to link behaviour with other welfare assessments [5,6]. In this context, zoo welfare researchers have redoubled their efforts to combine traditionally used health indicators (e.g., haematology, serum biochemistry) and physiological indices (e.g., hypothalamic–pituitary–adrenal, HPA activation) with measurements of behaviour [7,8]. This approach is useful to characterize activity patterns and study different behaviours, and allows making management recommendations, e.g., [9,10]. Additionally, the use of video technology is particularly useful for nocturnal and/or elusive species [11].

Glucocorticoids are another central component of stress responses, frequently used to evaluate the impact of stressful situations and the welfare of animals under human care [12]. The concentration of glucocorticoids (or their metabolites) can be measured in various body fluids or excreta [13,14]. Above all, faecal samples offer the advantage of an easy stress-free collection without the need for animal handling, and measured metabolites reflect adrenocortical activity over longer periods. For this reason, there has been an increase in the analysis of faecal glucocorticoid metabolites in zoos and wildlife facilities, e.g., [15,16,17,18,19,20,21].

Zoos or rescue centres, where wild animals are normally under human care, are considered a major component of ex situ conservation, and this working context may involve further management procedures to support conservation actions (e.g., translocation). In this regard, conservation interventions are a stressful challenge with different steps such as capture, veterinary check, transport, etc. [22,23]. Moreover, regular veterinary checks are part of the preventive medicine programs required by different zoo associations [24,25,26] and constitute a routine monitoring activity to determine the degree of commitment to animal welfare.

In this sense, studying the zoo-housed lesser anteater (*Tamandua tetradactyla)* reactivity to management procedures would be useful to support future management decisions. Therefore, we utilized validated, non-invasive methodologies to evaluate the behavioural and physiological stress responses in this species [27,28,29].

*Tamandua tetradactyla* (Mammalia: Pilosa: Myrmecophagidae) is a medium-sized anteater species endemic to South America. The great attractiveness of lesser anteaters has stimulated zoos to intensify their efforts to maintain them under human care, although many aspects of their biology remain to be elucidated [30,31]. Briefly, this semi-arboreal and solitary species exhibits a low metabolic rate associated with a low-energy diet, consisting primarily of social ants and termites. This species is described as nocturnal to diurnal–crepuscular, being active for approximately eight hours per day [32,33,34].

We presumed that zoo-housed *T. tetradactyla* individuals exhibited seasonal variations in stress levels, a common phenomenon in mammals [35,36]. Although housing conditions provided some protection from environmental challenges, the studied individuals were exposed to natural conditions of photoperiod, temperature and humidity. No information is available about seasonal changes in behaviour or adrenocortical activity in this species*;* which deserves particular attention considering several characteristics such as low body weight (<10 kg), low average body temperature (35 °C), low thermo-neutrality limit (23 °C), insectivorous diet, and general low metabolism and functions that may strongly influence its rhythmicity [37].

This study was developed considering the framework of the Reactive Scope Model proposed by Romero et al. [38] as well as other reports [39,40], focusing particularly on predictive and reactive homeostasis (i.e., in our study, seasonal stress changes and transport or veterinary check effect on stress responses, respectively). Reactive Homeostasis is the range of the concentrations/levels needed to respond to unpredictable or threatening environmental changes. The model predicts that above the predictive homeostasis range (e.g., seasonal changes) is the reactive homeostasis range, which represents physiological parameter values necessary to maintain homeostasis after an unpredictable event that threatens homeostasis. This model is relevant for understanding the stress response of wild animals under human care since they are normally kept under seasonal environmental changes.

The main purpose of this study was to non-invasively monitor behavioural and adrenocortical stress responses to management procedures of zoo-housed *T. tetradactyla*. Specifically, the effect of (1) season, (2) veterinary examination and (3) transport on activity budgets and faecal glucocorticoids were examined. Evaluating stress responses not only contributes to understanding the effects of different procedures but also ultimately leads to the development of improved management strategies. Furthermore, considering that the measurement of these indices is still far from straightforward in free-ranging wildlife, zoo-derived data have enormous potential not only to elucidate wild animal responses to captivity but also to improve normative data to enhance the precision of studies on wild populations. Thus, the present study may not only be useful to captive care specialists but also to scientists working with wild lesser anteaters, which are listed as Threatened in Córdoba [41], Near Threatened in Argentina, and Least Concern by the IUCN Red List of Threatened Species, see details in: [42,43]. Furthermore, it is important to take into account and acknowledge potential confounding factors, such as season, and use a rigorous experimental design to ensure proper interpretation of results [12,36].

## 2. Materials and Methods

### 2.1. Studied Animals and Housing Conditions

Twelve adult *Tamandua tetradactyla* (henceforth referred to as ‘lesser anteaters’; *n* = 6 females, neither pregnant nor lactating, and 6 males) were studied at Córdoba Zoo (now called Biodiversity Park, 31°12.32′ S; 64°16.84′ W; Córdoba, Argentina). Lesser anteaters were at least two years old (i.e., they had reached sexual maturity) and had been maintained in captivity for a minimum period of two years prior to the commencement of the study, further details in [28]. They were housed in individual contiguous enclosures of similar dimensions. Enclosures were designed following housing recommendations for the species [30]. The individual surface area of each enclosure was 38 ± 1.9 m^2^ (minimum required: 15 m^2^) and the height was 1.8 m (front) and 2.8 m (rear). Enclosures contained a wooden shelter, several climbing structures (e.g., logs, stairs and a wire roof), plants, soil and wood substrate. Each tamandua had at least one individual of the opposite sex as a neighbour to ensure that all individuals had a similar exposure to potential hormonal or pheromonal stimuli of the opposite sex. The gates (5 × 5 cm wire mesh) that communicate contiguous enclosures allowed lesser anteaters to climb, as well as to have visual, olfactory, auditory and tactile (minimal) contact with their neighbours.

Daily cleaning routines of enclosures were performed between 9:00 and 12:00 h by zookeepers. Food and water were supplied in plastic feeders located in hanging logs once a day, at approximately 12:00 h. Lesser anteaters’ balanced diet consisted of a semi-liquid shake containing lactose-reduced whole powdered milk (La Serenísima^®^, Buenos Aires, Argentina, 15% of total dry weight), baby cereal (Nestum^®^, Nestlé, Villa Nueva, Córdoba, Argentina, 14%), balanced dog feed for puppies (Eukanuba^®^ Small Breed, Buenos Aires, Argentina 71%), drinking water (until reaching a semi-liquid consistency, 400–600 mL) and vitamin K (3 mg). The offered quantity varied (700–900 mL) depending on the energetic need of each lesser anteater, which was calculated following Dierenfeld and Graffam [44]. Additionally, following Eguizábal et al. [27], food-based environmental enrichment (e.g., ants, honey, mealworms, fruits, etc.) was offered every two days, between 09:00 and 17:00 h, and exposed in enclosures for 24–48 h. According to veterinary records, lesser anteaters showed good health and nutritional status.

### 2.2. Climatic Housing Conditions

Lesser anteaters were maintained under natural conditions of photoperiod, temperature and humidity. Study 1 was performed during winter 2016 (21 July–18 August) and summer 2017 (21 January–18 February); study 2 during winter 2016 (21–25 August) and summer 2017 (21–25 February); and study 3 during summer (27–30 January) and winter 2019 (28–31 July).

During study 1 and 2, sunrise and sunset values were 08:00 and 18:45 h for winter, and 06:55 and 20:10 h for summer; the average temperature was 12.8 and 22.3 °C for winter and summer, respectively; and average humidity was 70.9 and 65.1% for winter and summer, respectively. During Study 3, sunrise and sunset values were 06:45 and 20:15 h for summer, and 08:00 and 18:45 h for winter; the average temperature was 25.4 and 10.9 °C for summer and winter, respectively; and average humidity was 62.3 and 79.1% for summer and winter, respectively. Environmental data were obtained from the web [45] for photoperiod and monitored using a mercury thermometer for temperature and a digital hygro-thermometer for humidity.

Following the recommendations of the zoo’s veterinary staff, there was an external heat source (150W, E27, General Electric, Buenos Aires, Argentina) inside each shelter, which was automatically turned on when the ambient temperature was lower than 10 °C.

### 2.3. Studies of the Seasonal Stress Response to Veterinary Procedures and Transportation

#### 2.3.1. Study 1—Effects of Seasons

For this study, three females and three males were included, and repeated measurements of behavioural observations and faecal samples were performed on the same individuals during 29 consecutive days in winter and summer.

#### 2.3.2. Study 2—Effects of Veterinary Check over the Seasons

The veterinary check was coordinated with the technical zoo staff of biologists and veterinarians. For this study, three females and three males were included, and repeated measurements of behavioural observations and faecal samples were performed on the same individuals during winter and summer. These lesser anteaters were the same animals studied in Study 1. Prior to the beginning of the study, the lesser anteaters had been subjected to routine veterinary checks over the last few years, so they were used to being handled.

The veterinary check consisted of a general health assessment. According to veterinarians’ recommendations, this procedure started by removing food from the enclosures at 18:30 h, since fasting was essential for the measurement of some of the variables analysed (e.g., cholesterol concentration). The following day, lesser anteaters were manipulated with the help of two veterinarians and two zookeepers during 3–5 min per individual (from capture to release) between 11:00 and 12:00 h (this schedule was arranged following the indication of zoo-technicians and the requirements of biochemical laboratory protocols). Briefly, each lesser anteater was captured by hand by the zookeeper, placed in a supine position on a metallic examination table located outside the enclosure, and physically immobilized by restraining the limbs and head. After blood extraction from the coccygeal vein, body measurements (thorax, mid and posterior abdomen circumferences) and simultaneous examination of ectoparasites were rapidly carried out (See Supplementary Materials, Figure S1, for a picture of the procedure). Lesser anteaters were released into their enclosures immediately after manipulation and received their daily food ration one hour after having been captured for the veterinary check. Therefore, the entire veterinary check protocol consisted of the combined effect of fasting and manipulation, which extended for approximately 18 h in total. It was applied from 22–23 August 2016 (winter) and from 22–23 February 2017 (summer). Individuals were randomly evaluated during the seasons.

Clinical values for all measurements (biometry, parasitology, haematology and serum biochemistry) exhibited variations within the normal ranges for specimens in good health, data not shown; [31].

#### 2.3.3. Study 3—Effects of Transportation over the Seasons

Terrestrial transport was coordinated with the technical zoo staff of biologists and veterinarians and approved by the Secretary of Environment of Córdoba Province, given the responsibility of both institutions (zoo and environmental authorities) in the management of lesser anteaters. Six females and six males were included in this study. Different individuals were monitored in summer and winter. Three of the six lesser anteaters previously included in Studies 1 and 2 were used in summer, and the other three in winter. In addition, we incorporated six new individuals, three of which were included in the experiments in summer and the other three in winter. Prior to the beginning of the study, the lesser anteaters had been exposed to several routine transports between enclosures or to the veterinary hospital within the zoo (distance of 1 km on internal roads), so they were used to being handled and placed in transport boxes.

The transport consisted of a terrestrial transfer using individual transport boxes (0.5 × 0.5 × 1.0 m; made of wood and covered with double wire mesh 0.05 × 0.05 m). It started when the zookeeper captured each individual manually using safety gloves and placed it in the transport box (<1 min). Briefly, transport boxes were placed in an open pickup truck and the procedure was simultaneously applied in all lesser anteaters (See Supplementary Materials, Figure S2 for a picture of the procedure). Transport lasted three hours (speed 20–30 km/h) and was carried out within the city of Córdoba during the light phase of the natural photoperiod, both for summer and winter. In summer, transport took place between 16:00–19:00 h on 28 January 2019; and in winter between 15:00–18:00 h on 29 July 2019. In both cases, transport finished approximately 1 h before sunset. Rainy days and extreme temperatures were avoided. Five short stops (<1 min) were made during each transport to verify the general condition of the lesser anteaters by visual inspection. Water was offered ad libitum in a plastic trough attached to the inner side of the transport box. The procedure ended when the zookeeper captured each individual manually using safety gloves and released them back into their original enclosure (<1 min).

### 2.4. Experimental Design and Sample Collection for Evaluation of the Effects of Management Procedures

As the number of lesser anteaters available for the study was low, it was not possible to carry out a classical experimental design (control and treatment). Therefore, an ABA experimental design was established, in which each individual acted as its own control when subjected to an experimental treatment [46]. It was not possible to increase the sample size (number of animals) since *T. tetradactyla* individuals in other Argentinean zoos are kept under different environmental and husbandry conditions, making it impossible to compare the results. Furthermore, transferring additional lesser anteaters from other institutions to Córdoba Zoo was restricted by the limited number of suitable enclosures. Lesser anteaters were kept in the enclosures for a 50-day acclimation period before the beginning of each study.

Briefly, following Saudargas and Drummer [46], sampling began the previous day in order to obtain measurements of a basal state without intervention (A: previous), followed by a period when the disturbance was applied (B: disturbance), and ended the day after management procedure (A′: posterior).

Non-invasive methodologies were used to evaluate the effect of routine management procedures both at the behavioural and adrenocortical levels (stress responses to veterinary check and transport). Behaviour was monitored by video recordings (for details, see Section 2.5) and adrenocortical activity by analysis of faecal glucocorticoid metabolites (FGMs; see Section 2.6). In the case of adrenocortical activity, a time-delayed sampling was carried out taking into account a time lag of 24 h [27] that allows inferring the activity during the period of faeces formation. Additionally, this previous study revealed that concentrations were restored to the values prior to the stimulus (ACTH challenge) in the subsequent sample, so it was not foreseen to consider a sampling effort greater than 24 h after the disturbance.

#### 2.4.1. Veterinary Check: Experimental Design and Sample Collection

**Figure 1:**
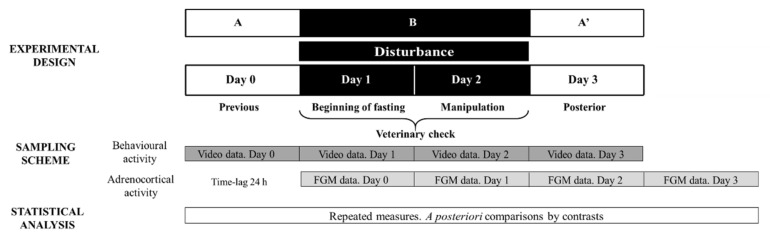
Representative diagram of the experimental and methodological approach used during Study 2 to evaluate veterinary check effect on behavioural and adrenocortical activity. FGM = faecal glucocorticoid metabolites. The ABA experimental design applied: day 0 (previous-stage A), days 1 and 2 (disturbance—stage B) and day 3 (posterior-stage A′).

Considering that the veterinary check procedure involved two potential stressors (fasting and manipulation), stage B consisted of two study days corresponding to these events. As the effect of fasting cannot be separated from that of manipulating the animal, the results obtained on both sampling days were averaged. Figure 1 depicts the ABA experimental design applied: day 0 (stage A), days 1 and 2 (disturbance—stage B) and day 3 (stage A′).

#### 2.4.2. Transport: Experimental Design and Sample Collection

**Figure 2:**
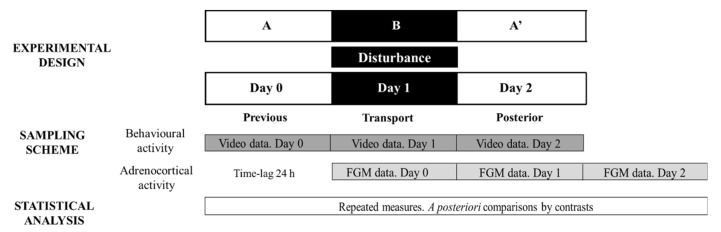
Representative diagram of the experimental and methodological approach used during Study 3 to evaluate transport effect on behavioural and adrenocortical activity. FGM = faecal glucocorticoid metabolites. The ABA experimental design applied: day 0 (previous-stage A), days 1 and 2 (disturbance—stage B) and day 3 (posterior-stage A′).

Figure 2 depicts the ABA experimental design applied and methodological approach to evaluate stress responses to transport: day 0 (stage A), day 1 (disturbance—stage B) and day 2 (stage A′).

### 2.5. Behavioural Analyses

Behaviour was continuously monitored by infrared video cameras located in each enclosure (HIKVISION Turbo HD-IR Turret Camera-DS 2CE56C2T IRM, Binjiang, China) and recorded by a digital video recorder (HIKVISION Turbo HD DVR-DS 7200 Series, Binjiang, China) located in the lab next to the enclosures. A behavioural evaluation was made by analysing video recordings during sampling days for each study and season.

Behaviour was sampled at 5 min intervals, using an adaptation of the instantaneous sampling method. Based on previous studies [29,47,48], we measured the behaviour (1 record/sample point) considering 15 s before and after sampling points. A total of 288 frequency records were obtained per individual each day [47,48]. It should be noted that it was not possible to record the behaviour of the lesser anteaters during the transport procedure in Study 3. Therefore, only 252 records were obtained for each individual during stage B (252 = 288 − 36 records, corresponding to 3 h of transport). In order to have the same number of records from all stages during Study 3, three hours of records from stages A and A′ (summer 16:00–18:59 h; winter 15:00–17:59) were removed from the analyses. Sunrise was considered as the beginning of the sampling day (minute 0), which ended moments before sunrise on the following calendar day (more details in Section 2.2). A previously developed ethogram was used for processing data [28]. Each record was classified as inactive or active, natural or abnormal. All observations were made by the same researcher (G.V.E.).

Several variables obtained from the daily behavioural records were evaluated. The total activity (TA) was calculated as the number of active records per day (considering all active behaviours; and natural activity (NAT) and abnormal activity (ABN) as the number of records of each category per day (considering all natural and abnormal active behaviours, respectively). Moreover, diurnal (DA) and nocturnal (NA) activities were calculated as the percentages of activity during natural light and dark phases, respectively. Considering that the number of sampling points of DA and NA varied in summer and winter due to the difference in natural photoperiodic phases, we compared both variables between seasons by calculating the percentages of activity. For instance, 70 active diurnal behaviour records per light phase correspond to 43.2% of the activity calculated as (70/162) × 100 = 43.2) in summer, and to 54.3% of the activity calculated as (70/129) × 100 = 54.3) in winter. The software ActogramJ [49] was used to build actograms from daily activity/inactivity records. They represent the way that the individuals’ activity is distributed throughout the day and allow obtaining the animals’ activity pattern. Actograms were also used to calculate variables associated with the animals’ activity cycle: acrophase (hour of peak of activity), beginning and end of activity (hour). All three variables were expressed as minutes since sunrise, with the latter representing minute 0.

### 2.6. Faecal Glucocorticoid Metabolite (FGM) Measurements

Adrenocortical activity was assessed using hormonal analyses. All fresh faeces were collected between 08:00 and 17:00 h during each sampling period. The number of collected faecal samples was 274 for Study 1 (20–36 samples per season and animal = 40–72 samples/animal); 41 for Study 2 (6–8 samples/animal); and 36 for Study 3 (6 samples/animal).

Each individual sample was immediately frozen at −20 °C and stored until processing for steroid analysis. Faecal glucocorticoid metabolites (FGMs) were extracted by adding 5 mL methanol/water (80%) to a portion (0.5 g) of each well-homogenized sample [50]. After shaking (2 min) and centrifugation (15 min; 3000 G), an aliquot (0.5 mL) of the supernatant was separated for analysis. The extracts were evaporated at 60 °C, shipped to Austria and re-suspended in methanol/water (80%) and dissolved in enzyme immunoassay (EIA) buffer. All measurements were run in duplicate with an 11-oxoaetiocholanolone EIA, as described by Möstl et al. [51]. This assay was previously successfully validated for *T. tetradactyla* [27]. The sensitivity of the EIA was 4 ng/g faeces. Inter-assay coefficients of variation (CV) for a low and high concentration pool sample, respectively, were 11.2 and 7.8% in Study 1 and 2, and 1.2 and 4.5% in Study 3. Intra-assay CV was always below 10%.

### 2.7. Statistical Analyses

For statistical analysis of Study 1, season (winter and summer) was included as a fixed term, and sex and individual were random factors. For Study 2 and 3, models with management procedure stage (A, B and A′; veterinary check or transport) and season (winter and summer) as fixed terms were run; sex and individual were included as random factors. The behavioural variables TA, NAT and ABN were analysed using mixed general linear models (MGLM) for all studies, and a Poisson error distribution was assumed. On the other hand, DA and NA were analysed using mixed linear models (MLM). When data did not meet a normal distribution, a square root transformation was applied. Variables associated with the animals’ activity cycle were also analysed using MGLM with the assumption of a Poisson error distribution. Adrenocortical data were transformed to a decimal logarithm to meet normal distribution and analysed using MLM.

For a posteriori tests, Fisher’s test was applied when the statistical analysis showed a *p*-value ≤ 0.05 in Study 1. On the other hand, to reveal the potential effect of seasonal management procedures and answer specific questions from these studies, planned comparisons, often termed contrasts; [52] were used for the data obtained during Study 2 and 3. When a bifactorial effect of routine management procedure and season was detected, contrasts were established for the comparisons: A*w* vs. B*w* and A*s* vs. B*s* (*w* = winter and *s* = summer; question: was there an effect of management procedure within each season?), A*w* vs. A′*w* and A*s* vs. A′*s* (question: were baseline and posterior states to the management procedure similar within each season?), and B*w* vs. B*s* (question: were there differences in the seasonal values achieved during the management procedure?). If no bifactorial effect was detected, the uni-factorial effect of routine management procedure was evaluated applying the following contrasts: A vs. B and A vs. A′. For uni-factorial effect of seasons, Fisher’s a posteriori test was applied.

For all cases, normality was verified using the modified Shapiro–Wilks test and variance homogeneity using Levene’s test. All analyses were performed using InfoStat [53]. Values are reported as the mean ± SEM unless otherwise noted, and the significance level was 5% for all tests.

## 3. Results

### 3.1. Study 1—Effects of Seasons

Seasonal variation was detected in some behavioural variables and adrenocortical activity (Table 1).

**Figure 3:**
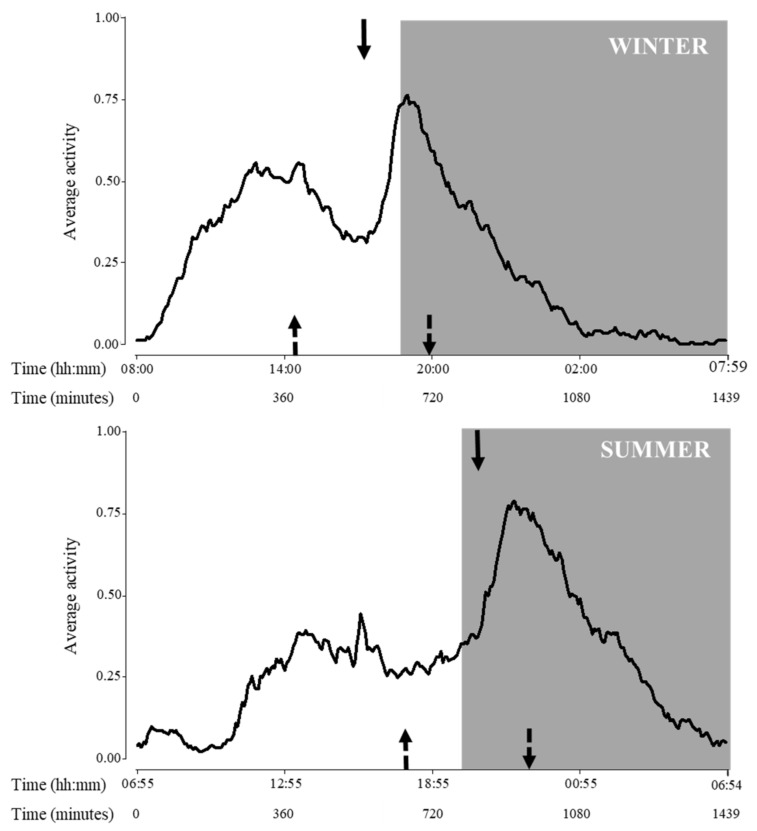
Activity pattern during the day of adult *Tamandua tetradactyla* (*n* = 3 females and 3 males) exposed to semi-controlled conditions, during winter (upper panel) and summer (lower panel). Behaviour was recorded every 5 min (288 records/day/individual) during 29 consecutive days for each season, and the average activity was calculated. Arrows represent activity cycle variables during each season (top arrow: acrophase, upward-pointing dotted arrow: beginning of activity, and downward-pointing dotted arrow: end of activity). Significant differences were found (*p* values in the text). Light phase: white background; dark phase: grey background. Beginning of X axis corresponds to sunrise for each season due to natural photoperiodic variations. Time is expressed both in hours and minutes and in minutes since sunrise.

Regarding activity cycle, variations due to seasons were detected for all variables (*p* < 0.0001 for all cases, F_1,10_ = 428.8, 320.7 and 221.0, for beginning, acrophase and end of activity, respectively; Figure 3).

### 3.2. Study 2—Effect of Veterinary Check

#### 3.2.1. Behavioural Response to Veterinary Check

**Figure 4:**
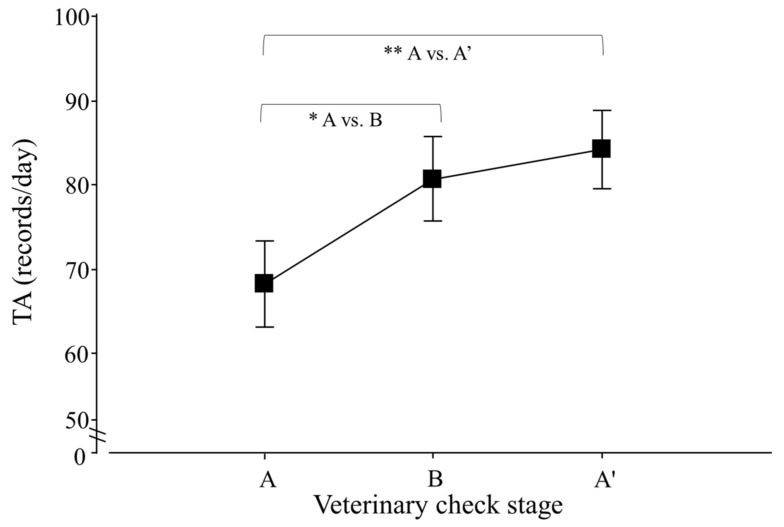
Total activity (TA) per day of adult *Tamandua tetradactyla* (*n* = 3 females and 3 males) exposed to semi-controlled conditions, in response to veterinary check (applied in B) during winter and summer. Data from winter and summer were pooled because there was no effect of season. The ABA experimental design applied: day 0 (previous-stage A), days 1 and 2 (disturbance—stage B) and day 3 (posterior-stage A′). Behaviour was recorded every 5 min (288 records/day/individual), and the number of activity records per day during each stage was calculated. Asterisks indicate significant differences between stages for all individuals (*p* values in the text). Results are expressed as the mean ± SEM.

Regarding the total activity (TA) of lesser anteaters, only an effect of veterinary check, but not of season, was detected (*p* < 0.0001; F_2,30_ = 14.5), and contrasts revealed that A ≠ B and A ≠ A′ (*p* < 0.0001 for both cases; F_1,25_ = 16.6 and 26.9; respectively; Figure 4).

**Figure 5:**
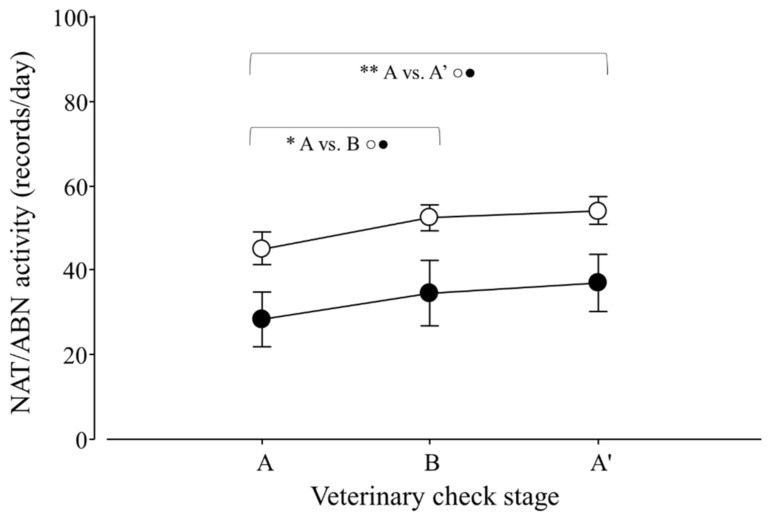
Natural (NAT; ○) and abnormal activity (ABN; ●) per day of adult *Tamandua tetradactyla* (*n* = 3 females and 3 males) exposed to semi-controlled conditions, in response to veterinary check (applied in B) during winter and summer. Data from winter and summer were pooled, because there was no effect of season. The ABA experimental design applied: day 0 (previous-stage A), days 1 and 2 (disturbance—stage B) and day 3 (posterior-stage A′). Behaviour was recorded every 5 min (288 records/day/individual), and the number of activity records per day during each stage per season was calculated. Asterisks indicate significant differences between stages for all individuals (*p* values in the text). Results are expressed as the mean ± SEM.

Variations in natural and abnormal activity due to the veterinary check were detected (*p* = 0.0105 and 0.0036; F_2,30_ = 6.8 and 7.7, respectively). For both variables, contrasts revealed that A ≠ B (*p* = 0.0103 and 0.0076; F_1,25_ = 6.6 and 7.1; respectively; Figure 5) and A ≠ A′ (*p* = 0.0021 and 0.0003; F_1,25_ = 9.5 and 13.0; respectively; Figure 5). Moreover, an effect of seasons on ABN was detected (winter 26.7 ± 13.0 > summer 22.7 ± 11.1 records/day; *p* = 0.0094; F_1,30_ = 7.7).

No variations due to veterinary check or season were detected when evaluating diurnal and nocturnal activity. When analysing the activity cycle, an effect of veterinary check (*p* < 0.0001; F_2,30_ = 68.2; contrasts in Table 2) and season (winter 377.7 ± 49.5 < summer 670.9 ± 65.6 min; *p* < 0.0001; F_1,30_ = 1429.7) was found for beginning of activity. There were variations due to the interaction of veterinary check and season for acrophase and end of activity (*p* < 0.0001 for both cases; F_2,30_ = 27.3 and 27.2, respectively; contrasts in Table 2).

#### 3.2.2. Adrenocortical Response to Veterinary Check

No significant differences were found in FGM concentrations due to the veterinary check. However, analysis revealed seasonal changes in FGM levels (winter 1.9 ± 0.2 < summer 7.2 ± 0.9 μg/g; *p* < 0.0001; F_1,25_ = 44.8).

### 3.3. Study 3—Effect of Transportation

#### 3.3.1. Behavioural Response to Transportation

**Figure 6:**
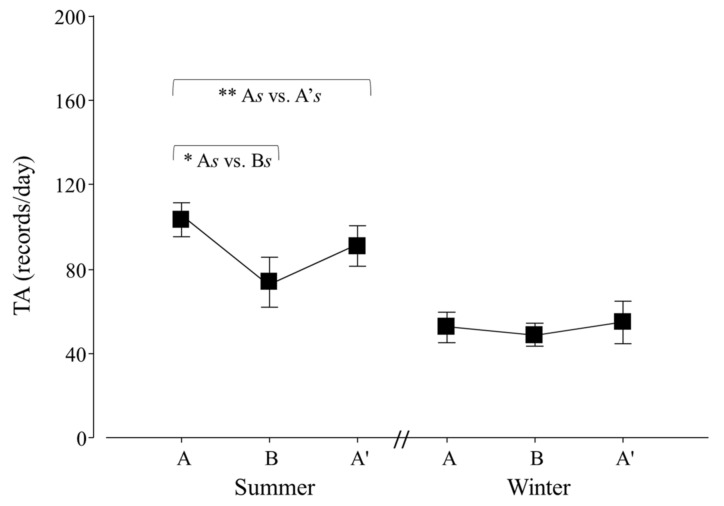
Total activity (TA) per day of adult *Tamandua tetradactyla* (*n* = 6 females and 6 males) exposed to semi-controlled conditions, in response to seasonal transports (applied in B) during summer and winter. The ABA experimental design applied: day 0 (previous-stage A), days 1 and 2 (disturbance—stage B) and day 3 (posterior-stage A′). Behaviour was recorded every 5 min (252 records/day/individual), and number of activity records per day during each stage per season was calculated. Asterisks indicate significant differences between stages during seasons for all individuals (* A*s* ≠ B*s*, ** A*s* ≠ A′*s*; *p* values in the text). Results are expressed as the mean ± SEM.

An effect of seasonal transport on the total activity (TA) of lesser anteaters was detected (*p* = 0.0403; F_2,30_ = 3.6). Contrasts showed that A*s* ≠ B*s*, A*s* ≠ A′*s* and B*s* ≠ B*w* (*p* = 0.0001, 0.0280 and 0.0056; χ^2^_1,25_ = 29.2, 4.8 and 8.0; respectively; Figure 6).

**Figure 7:**
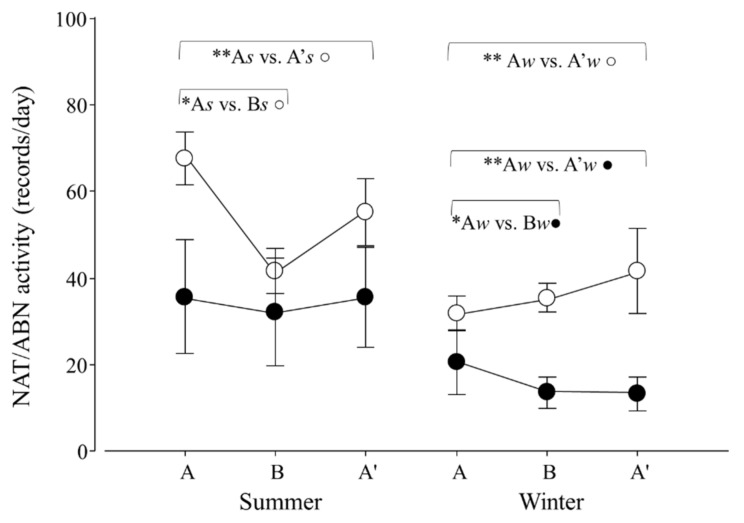
Natural (NAT, ○) and abnormal activity (ABN, ●) per day of adult *Tamandua tetradactyla* (*n* = 6 females and 6 males) exposed to semi-controlled conditions, in response to seasonal transports (applied in B) during summer and winter. The ABA experimental design applied: day 0 (previous-stage A), days 1 and 2 (disturbance—stage B) and day 3 (posterior-stage A′). Behaviour was recorded every 5 min (252 records/day/individual), and number of activity records per day during each stage per season was calculated. Asterisks indicate significant differences between stages during seasons for all individuals (* A*s* ≠ B*s ○*, ** A*s* ≠ A′*s* ○, ** A*w* ≠ A′*w ○*, * A*w* ≠ B*w ●*, ** A*w* ≠ A′*w* ●; *p* values in the text). Results are expressed as the mean ± SEM.

When considering natural and abnormal activity, variations in both variables in response to seasonal transport were found (*p* = 0.0001 and 0.0396; F_2,30_ = 12.3 and 3.6). For NAT, contrasts revealed that A*s* ≠ B*s*, A*s* ≠ A′*s* and A*w* ≠ A′*w* (*p* = 0.0001, 0.0058 and 0.0057; χ^2^_1,25_ = 36.5, 7.6 and 7.6, respectively; Figure 7). For ABN, contrasts revealed that A*w* ≠ B*w* and A*w* ≠ A′*w* (*p* = 0.0033 and 0.0020; χ^2^_1,25_ = 8.6 and 9.5; respectively; Figure 7).

**Figure 8:**
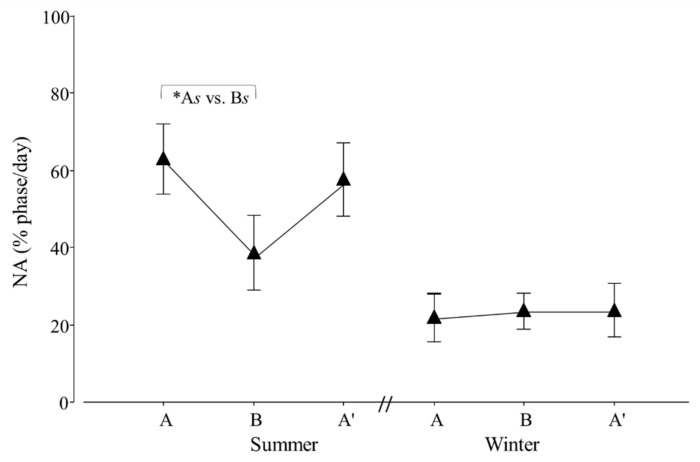
Percentage of nocturnal (NA) activity per day of adult *Tamandua tetradactyla* (*n* = 6 females and 6 males) exposed to semi-controlled conditions, in response to transport (applied in B) during summer and winter. The ABA experimental design applied: day 0 (previous-stage A), days 1 and 2 (disturbance—stage B) and day 3 (posterior-stage A′). Behaviour was recorded every 5 min (252 records/day/individual), and the percentage of activity during dark phase per day during each stage was calculated. Asterisk indicates a significant difference between stages during summer for all individuals (* A*s* ≠ B*s*; *p* value in the text). Results are expressed as the mean ± SEM.

Regarding diurnal and nocturnal activity, an effect of seasonal transport was detected for NA (*p* = 0.0332; F_2,20_ = 4.1). Contrasts revealed that A*s* ≠ B*s* (*p* = 0.0309; χ^2^_1,20_ = 5.4; Figure 8).

When analysing the data of the animals’ activity cycle, there was a bi-factorial effect of seasonal transport on beginning, acrophase and end of activity (*p* = 0.0017, 0.0002 and 0.0001; F_2,30_ = 7.9, 11.3 and 14.0, respectively; contrasts in Table 3).

#### 3.3.2. Adrenocortical Response to Transport

No significant differences in FGM concentrations during winter and summer transports were observed (A = 1.4 ± 0.1; B = 1.7 ± 0.3; A′ = 1.7 ± 0.3 μg/g); no statistical variation was detected due to seasons.

## 4. Discussion

Non-invasive methodologies were used to assess seasonal behavioural and adrenocortical responses to management procedures in adult *Tamandua tetradactyla* under human care. In Study 1, levels of stress responses considering the reactive scope model proposed by Romero [38] were analysed, aiming to characterize the predictive range. We consider that the values obtained reflect undisturbed situations over 29 consecutive days, in which each individual faced normal activities and housing conditions. Findings indicated that the seasonality hypothesis was: (a) confirmed for some variables, such as nocturnal activity, activity cycle, and adrenocortical activity; and (b) rejected for other behavioural variables, such as total activity, diurnal, natural, and abnormal activities (repetitive locomotion). These results served as a basis to conduct further analyses in Studies 2 and 3, which aimed at comparing the stress response to management procedures according to seasons. Findings indicated that: (a) animals responded to management procedures at a behavioural level; (b) no changes due to procedures were detected at the adrenocortical level; and (c) responses to management procedures may exhibit seasonality.

A biological rhythm is a behavioural or physiological attribute that changes over time on a predictable cycle. Many environmental cues/factors occur with regularity, for example, photoperiodic changes. The ability to predict these changes, whether daily or annual, is adaptive [36]. This study showed that zoo-housed lesser anteaters exhibited similar levels of total activity over the seasons, but they were more active in summer nights than in winter nights. The latter could be related to the seasonal variations in photoperiod in Córdoba, Argentina (approximately 14 vs. 10 h of light/day, respectively). Zoo-housed lesser anteaters may use photoperiodic information (e.g., hour of light/day) to organize activities and predict environmental changes, such as cooler periods of day in summer. In this sense, the most challenging environmental factor for lesser anteaters may be ambient temperature because of their physiological characteristics of low metabolic rate and thermo-neutrality level [54]. Thus, we consider that an additive effect of temperature may also explain changes in some behavioural variables, as winter nights in Córdoba (when the ambient temperature is approximately 10 °C below their thermo-neutrality) may be stressful for this species. There are reports of seasonality in related species, such as giant anteaters, *Myrmecophaga tridactyla*; [55,56]. Particularly, Di Blanco et al. [56] showed that wild giant anteaters spent more hours active at night during the summer compared to other seasons and concluded that seasonal shifts in daily activity highlighted the importance of thermoregulation as a selective factor in this species. Perhaps, the influence of seasons (photoperiod and temperature) on activity synchronization persists in lesser anteaters under human care.

Wild individuals may accommodate their activity pattern to local conditions. Therefore, it was assumed that zoo-housed lesser anteaters could exhibit flexibility, predicting that individuals would change their activity pattern according to environmental conditions [28]. Indeed, studied lesser anteaters spent more time active on warm summer nights than on cold winter nights, which would indicate an effect of temperature on activity synchronization. Although humidity also naturally varied in this study, variations (less than 5%) among seasons were not relevant. Nevertheless, this is an environmental parameter to be considered in the modulation of the stress response, since warmer days with high levels of humidity would be a challenge for this species, which has a low thermo-neutrality limit (23 °C).

Regarding the temporal dynamics of lesser anteaters’ activity, clear differences among seasons were found in the activity cycle analyses: beginning, peak and end of activity were delayed during summer compared to winter. Particularly, considering acrophase, lesser anteaters reached the highest seasonal levels of activity approximately 9 and 14 h after sunrise in winter and summer, respectively. In this sense, findings showed that seasons affected lesser anteaters’ dynamics, with peaks of activity occurring at 17:00 h in the light phase during winter, and at 21:00 h in the dark phase during summer. The animals studied seemed to synchronize their activities to avoid potentially deleterious environmental conditions, such as cold nights during winter and hot days during summer.

Seasonal analyses also showed that some variables were not sensitive to seasons, such as total activity, diurnal, natural, and abnormal activity (repetitive locomotion). There are several possible explanations for this lack of seasonality. First, when considering diurnal activity, although results suggest that winter and summer light phases were not thermally stressful to lesser anteaters, the *p*-value (0.0812) for seasonal differences is in favour of rejecting the null hypothesis (seasonality). Second, total activity as well as natural activity includes several behaviours, such as feeding or locomotion, which can be associated with different motivations or needs and may respond differently to internal and/or external sources of variations. This uncontrolled variation in daily repertories could therefore negatively affect the statistical analysis. Further studies on each behaviour separately could bring to light how animals respond to seasons. Third, repetitive locomotion (also known as pacing) has been proved to be a fixed abnormal behaviour among captive individuals, which is extremely hard to tackle or modify [57]. Generally speaking, it is important to note two points: (1) in the context of the experimental design, future studies should consider collecting data during more than one day post-event so that a latency to return to pre-event levels of activity could be better determined, since A was often different from A′; (2) in the context of the reactive scope model, these variables should be analysed using the basic non-seasonal species model, although studies with a higher number of experimental units may change the response pattern of *T. tetradactyla* to seasonality.

Seasonal adrenocortical activity evaluated by faecal glucocorticoid metabolite variations indicated that lesser anteaters exhibited higher levels during summer compared to winter. Studied animals presumably had different concentrations of glucocorticoids circulating in the blood throughout the seasons. Several hypotheses about why wild animals exhibit seasonality in adrenocortical activity have been proposed, e.g., see [35]. Adrenocortical activity levels of zoo-housed lesser anteaters may have been higher in summer, mediating their greatest behavioural needs during this season. Therefore, faecal glucocorticoid metabolites could provide information about behavioural activities of *T. tetradactyla* individuals facing seasonal environmental challenges. Considering that glucocorticoids are involved in the mobilization of body energy, among other functions; see [58], this study provides evidence that lesser anteaters displayed a strategy for efficient energy use and thermoregulation. Faecal glucocorticoid metabolite measurement has been reported as a good indicator of thermoregulatory demands in ursids. An increase in adrenocortical activity was detected in wild Grizzly and American black bears while ambient temperature descended during autumn [59]. This is an opposite pattern to that of the zoo-housed lesser anteaters studied here, which showed the lowest values during winter. The interaction of physiological strategies and environmental challenges may explain the different responses in these species, but additional research is needed to confirm this.

Our results may contribute to ongoing discussions about the role of glucocorticoids as promoters of fitness during different environmental conditions, e.g., [60,61]. Because our experimental conditions impeded reproduction due to individual housing, the variations in glucocorticoid levels of lesser anteaters probably indicated primarily a variation in energy expenditure rather than breeding activities, which were possible in other studies that housed males and females together, e.g., [21,62].

Veterinary check elicited changes in the total activity of lesser anteaters, causing higher levels of TA, NAT and ABN during and after the procedure. Furthermore, all parameters of the activity cycle were affected; animals began activity earlier in response to the procedure, and they showed a delayed peak and end of activity in response to veterinary check during the summer. This could be related to the manipulation for blood collection, which was carried out outside normal active hours of lesser anteaters (14:00–20:00 h in winter and 18:00–23:00 h in summer) and perhaps acted as a disruptor when animals were inactive. An alternative explanation for the increased activity could be a reaction to fasting (i.e., animals were hungry, searching for food) and/or to manipulation (i.e., animals were alert after being captured for blood extraction). Future studies focusing on which particular behaviours are increased in response to veterinary checks may help clarify this point.

Although the veterinary check elicited a behavioural response, the values reported for stage B seem to be within the predictive range determined during Study 1 (see Table 1). No adrenocortical response to veterinary check was detected. Behavioural changes triggered by a stimulus usually represent a first stress response, and the degree of activation of the associated physiological response may vary. In their review on plasma corticosterone levels in rats in response to natural and artificial stressors, Koolhaas et al. [63] found that stress physiological patterns depended, among other factors, on the stressor (sexual behaviour > novel cage > handling). In the context of reactive homeostasis, fasting and manipulation (veterinary check) did not affect faecal glucocorticoid metabolites of zoo-housed lesser anteaters, and adrenocortical levels were sufficient to cope with this type of ‘stressful’ situation. However. it should be noted that lag time and spontaneous daily defecation vary among species. Some may defecate several times per day [64] while others, such as the lesser anteater, only once daily [27]. Reports from species that allowed collection of several samples during a 24 h period showed an FGM peak of 10–12 h after management procedures [65,66]. We can therefore not exclude the possibility that the short-term increase in adrenocortical activity in response to the management procedures could not be detected in lesser anteaters because of the 24-h accumulation of faecal glucocorticoid metabolites during stage B (disturbance day).

The animals’ perception of a stressor can affect their adrenocortical response. The lesser anteaters studied here had been subjected to routine veterinary checks over the last few years and were thus used to being handled. It is therefore possible that their positive or neutral previous experience contributed to attenuating their response. Although findings seem to indicate that veterinary checks only had a slight effect when considering both physiological and behavioural results, we cannot exclude the possibility that the animals perceived the procedure as a stressor. Nevertheless, several factors, such as previous negative handling experience, animal personalities or animals’ welfare status should be borne in mind when applying veterinary procedures in other facilities or other lesser anteaters, as they could lead to results different from those presented here.

In Study 3, terrestrial transport was applied on zoo-housed lesser anteaters. Several studies on wild animals exposed to transportation have reported that welfare was affected [67]. Most of these works, however, also included results collected at the new destination, therefore, assessing the cumulative effect of transport and the novel environment [68,69]; this was not the case for lesser anteaters because they were released back into their enclosures.

Changes in total activity were detected in response to this procedure during summer, showing that zoo-housed lesser anteaters reduced their total activity during the transportation day. Considering that some studies have reported a reduction in the activity level of animals subjected to transport and relating this decrease to a high energetic demand [70,71], transportation seems to be a challenging procedure for lesser anteaters. Perhaps, differences detected later (A′ higher than B, but still lower than A) indicate that animals were still recovering after the procedure. In addition, the reduction in NA during the day of transportation in summer showed a similar pattern, indicating perhaps that lesser anteaters reduced activities over the night to restore energy by resting. Besides, transportation affected activity cycle of zoo-housed lesser anteaters. The timing of the activity shifted and the responses were different in summer and winter. In summer, the beginning and peak of activity were earlier on the disturbance day because transport started at 16:00 h and the animals usually began their activities at 18:00 h. The day after transport, the animals began activity later but restored the acrophase parameter. Both changes in the activity cycle could indicate that after the procedure, the animals still needed to restore their energy in the short term after transport had caused a partial energetic demand, e.g., [72], being inactive for an extended period. In winter, transport led to an increased value of acrophase (this means that activity peaked later than in stage A), possibly because it was performed during 3 of the 4 h during the light phase when animals were usually active. In sum, the difference in the beginning of activity detected only in summer (B < A) is expected since lesser anteaters were not active when transport took place; the opposite is true when transport took place in winter and all individuals were already active. There was an apparently opposite effect of transport on the peak activity over the seasons, causing a delay in winter and peaking earlier in summer. In both seasons, the end of activity was not affected by transportation, indicating that disruption during the day of transportation would be mild for these individuals.

The expression of natural behaviours (e.g., exploration, socialization, etc.) gives animals control over their environment and allows them to alleviate their stress responses [73,74,75,76]. The reduced natural activity during transport day in summer observed in *T. tetradactyla* could therefore indicate less control over their environment or a reduced ability to face the challenging situation. Additional studies focusing on which particular behaviours are reduced in response to transportation are needed. Similarly, more studies are necessary to understand changes in abnormal behaviours, particularly different responses detected over the seasons (only a reduction during winter, which may be considered positive for these animals). It has been reported in other studies that stereotypical behaviours change after transportation, e.g., increase in stereotypical behaviours after transportation to new places; [72]. Although the studied lesser anteaters were not trained, they may have been habituated because transport is a routine procedure. Dembiec et al. [70] showed that tigers without previous experience with a transport procedure exhibited a higher increase in cortisol levels after transport than experienced individuals (482% vs. 158% above baseline, respectively). Similarly, training has proven useful to mitigate the stress response to transport in donkeys [77].

In the context of the Reactive Homeostasis Model, transportation elicited a behavioural response in lesser anteaters (decreased natural activity during summer, changes in diurnal and nocturnal activity, and alterations of activity cycle), but values seem to be within the predictive range reported for Study 1. This could suggest that transport was not a strong stressor, as it did not affect adrenocortical activity. In this context, changes in FGM levels were expected during stage B for the summer because the natural activity was reduced and this variable was negatively correlated with FGMs in a previous study on lesser anteaters [28]. It is possible that the procedure did not generate a large demand on adrenocortical function. On the other hand, FGM levels seem to be outside the predictive range reported in Study 1. Perhaps, internal and external impacts (such as age and life history) on individual adrenocortical function in Study 3 may explain that values of FGMs were not included in the predictive range. For instance, Palme [12] pointed out that the “individual” plays a crucial role in all aspects of stress research and individual differences have been observed in all parts of the HPA axis and may be also related to glucocorticoid metabolism and excretion. These differences may be of genetic origin but may also be acquired during life. Thus, we think that in the context of the reactive homeostasis model, more attention should be directed towards individual effects on this hormonal variable than on behavioural ones in lesser anteaters,

From the perspective of the stress response, management procedures only affected the behavioural activities of zoo-housed lesser anteaters, which would indicate they only had a slightly negative effect on the animals’ welfare state. Although changes were recorded in several variables of the overall activity and its dynamics throughout the day, no clear pattern of response to management procedures was detected. As for the physiological response, the applied procedures may have led to some changes in the HPA axis that remained undetected due to the sampling scheme, i.e., the low-level response may have been diluted by normal concentrations over the prolonged window (~24 h) of faecal measures.

The framework of the reactive scope model proposed by Romero et al. [38] was not only useful to design the experimental study but also to understand our results. Although winter and summer ranges were not mutually exclusive, the differences revealed were useful to analyse the stress response of zoo-housed lesser anteaters. However, the low number of lesser anteaters under human care in Argentina has been a weakness in this analysis, and additional evidence is needed to improve the usefulness of the predictive ranges reported in Table 1, and the responsive ranges mentioned in Table 2 and Table 3. On the other hand, behavioural changes in response to management procedures were detected, but these values were not above the range of predictive homeostasis as predicted by the model. Although this does not invalidate the model, perhaps some factors such as those mentioned above (e.g., previous experience) could explain why the measurements did not present values outside of the predictive range. Beyond this limitation, this study contributes to a better understanding of the stress response in wild animals under human care. The data generated may be useful for ecologists studying stress in free-ranging lesser anteaters.

## 5. Conclusions

Zoo-housed lesser anteaters under natural variations of photoperiod, temperature and humidity showed: (a) seasonality in their nocturnal activity and daily adrenocortical activity; and (b) no seasonal changes in most other behaviours of their natural repertories and repetitive locomotion (the only stereotype exhibited). The animals also showed seasonality in their activity cycle, being active later in summer than in winter.

Veterinary and transport procedures only triggered a behavioural response, which was greater during summer and did not elicit significant changes in adrenocortical activity. The effect of the procedures on lesser anteaters was similar at the activity cycle level, with acrophase changing seasonally.

By applying non-invasive techniques, this study demonstrated the importance of monitoring animals at different levels of the biological system (behavioural and hypothalamic–pituitary–adrenal axis). The results suggest that the management procedures tested here did not represent a major challenge to homeostasis. Nevertheless, we recommend performing them during winter to minimize reactivity at the behavioural level, but further studies on the stress responses of lesser anteaters are suggested to increase the sample size.

## Figures and Tables

**Table 1:** Behavioural and adrenocortical activity in adult *Tamandua tetradactyla* individuals exposed to semi-controlled conditions during winter and summer.

Activity	Variable	Season				Statistics
		Winter	Min-Max	Summer	Min-Max	
Behavioural	TA (records/day)	80.5 ± 6.3	63.6–98.6	85.9 ± 5.7	71.4–105.1	*p* = 0.3353; F_1,10_ = 1.0
	NAT (records/day)	44.5 ± 2.7	38.4–55.4	49.7 ± 2.9	39.2–56.3	*p* = 0.2208; F_1,10_ = 1.7
	ABN (records/day)	33.2 ± 7.5	8.4–57.3	36.2 ± 8.1	15.5–57.3	*p* = 0.3972; F_1,10_ = 0.8
	DA (%)	37.2 ± 11.5	5.0–72.1	27.5 ± 8.1	6.7–50.5	*p* = 0.0812; F_1,5_ = 4.8
	NA (%)	20.5 ± 5.8	2.9–39.7	32.7 ± 6.0	16.0–52.7	*p* = 0.0008; F_1,5_ = 51.6
Adrenocortical	FGMs (μg/g)	2.8 ± 0.2	1.95–3.46	5.1 ± 0.9	2.38–8.78	*p* = 0.0337; F_1,5_ = 8.4

TA: total activity, NAT: natural activity and ABN: abnormal activity (repetitive locomotion), DA: diurnal activity and NA: nocturnal activity; FGMs: faecal glucocorticoid metabolites as μg/g fresh faeces. Results are expressed as the mean ± SEM; and minimum (Min) and maximum (Max) values are reported.

**Table 2:** Effect of seasonal veterinary check on activity cycle of adult *Tamandua tetradactyla* maintained in semi-controlled conditions.

Variable	Season	Veterinary Check			Contrasts		
Beginning of activity		A	B	A′	A ≠ B		A ≠ A′
		589 ± 93	491 ± 71	493 ± 85	*p* < 0.0001; χ^2^ = 100.6		*p* < 0.0001; χ^2^ = 97.7
Acrophase		A	B	A′	B*w* ≠ B*s*	A ≠ B	A ≠ A′
	Winter	566 ± 71	559 ± 78	535 ± 83	*p* < 0.0001; χ^2^ = 182.2	No differences	*p* = 0.0228; χ^2^ = 5.2
	Summer	682 ± 133	759 ± 83	819 ± 92		*p* < 0.0001; χ^2^ = 25.1	*p* < 0.0001; χ^2^ = 75.4
End of activity		A	B	A′	B*w* ≠ B*s*	A ≠ B	A ≠ A′
	Winter	738 ± 58	743 ± 89	708 ± 81	*p* < 0.0001; χ^2^ = 139.3	No differences	No differences
	Summer	826 ± 167	941 ± 90	981 ± 122		*p* < 0.0001; χ^2^ = 45.1	*p* < 0.0001; χ^2^ = 79.6

Timing is expressed in minutes since sunrise (minute 0). Significant uni- and bi-factorial contrasts between stages for each variable are presented, being *w* winter and *s* summer. Results are expressed as the mean ± SEM. Lesser anteaters were manipulated during 3–5 min per individual (from capture to release) between 11:00 and 12:00 h (in winter: 180–240 min after sunrise, and in summer 245–305 min after sunrise). The ABA experimental design applied: day 0 (previous-stage A), days 1 and 2 (disturbance—stage B) and day 3 (posterior-stage A′).

**Table 3:** Effect of seasonal transport on activity cycle of adult *Tamandua tetradactyla* maintained in semi-controlled conditions.

Variable	Season	Transport			Contrasts		
		A	B	A′	B*w* ≠ B*s*	A ≠ B	A ≠ A′
Beginning of activity	Winter	542 ± 77	553 ± 56	550 ± 59	No differences	No differences	No differences
	Summer	700 ± 83	665 ± 76	754 ± 14		*p* = 0.0203; χ^2^ = 5.4	*p* = 0.0005; χ^2^ = 121.0
Acrophase	Winter	642 ± 79	679 ± 43	657 ± 58	*p* = 0.0444; χ^2^ = 4.0	*p* = 0.0135; χ^2^ = 6.1	No differences
	Summer	907 ± 66	854 ± 87	937 ± 45		*p* = 0.0019; χ^2^ = 9.7	No differences
End of activity	Winter	788 ± 91	793 ± 81	785 ± 78	*p* = 0.0207; χ^2^ = 5.4	No differences	No differences
	Summer	1033 ± 116	1063 ± 107	1175 ± 62		No differences	*p* < 0.0001; χ^2^ = 551.0

Timing is expressed in minutes since sunrise (minute 0). Significant bi-factorial contrasts between stages for each variable are represented, being *w* winter and *s* summer. Results are expressed as the mean ± SEM. In summer, transport took place between 16:00–19:00 h (420–600 min after sunrise), and in winter between 15:00–18:00 h (545–725 min after sunrise). The ABA experimental design applied: day 0 (previous-stage A), days 1 and 2 (disturbance—stage B) and day 3 (posterior-stage A′).

## Data Availability

The data presented in this study are available upon request from the corresponding authors.

## Supplementary Materials

The following supporting information can be downloaded at: https://www.mdpi.com/article/10.3390/ani12010075/s1, Figure S1: Veterinary check of adult *Tamandua tetradactyla* during winter (23 August 2016) of Study 2. Panel A shows the moment in which blood extraction from Male #3 is carried out and Panel B in which abdomen circumference from Male #2 is determined. Photos by Franco Rios; Figure S2: Transport of adult *Tamandua tetradactyla* during Study 3. Panel A shows how animals were transported (Summer and Winter). Panel B shows Female #2 inside the box, previous to winter transport (29 July 2019). Photos by Gabina Eguizábal.
