# Supplementary material for: Non-Invasive Assessment of the Seasonal Stress Response to Veterinary Procedures and Transportation of Zoo-Housed Lesser Anteater (Tamandua tetradactyla)
Source: Animals (Basel). 2021 Dec 30;12(1):75. doi: 10.3390/ani12010075 (PMC8744720; doi:10.3390/ani12010075)
Supplement: Supplementary file 1 [file animals-12-00075-s001.zip › animals-1511770-supplementary.pdf]

animals
MDPI

Supplementary Materials

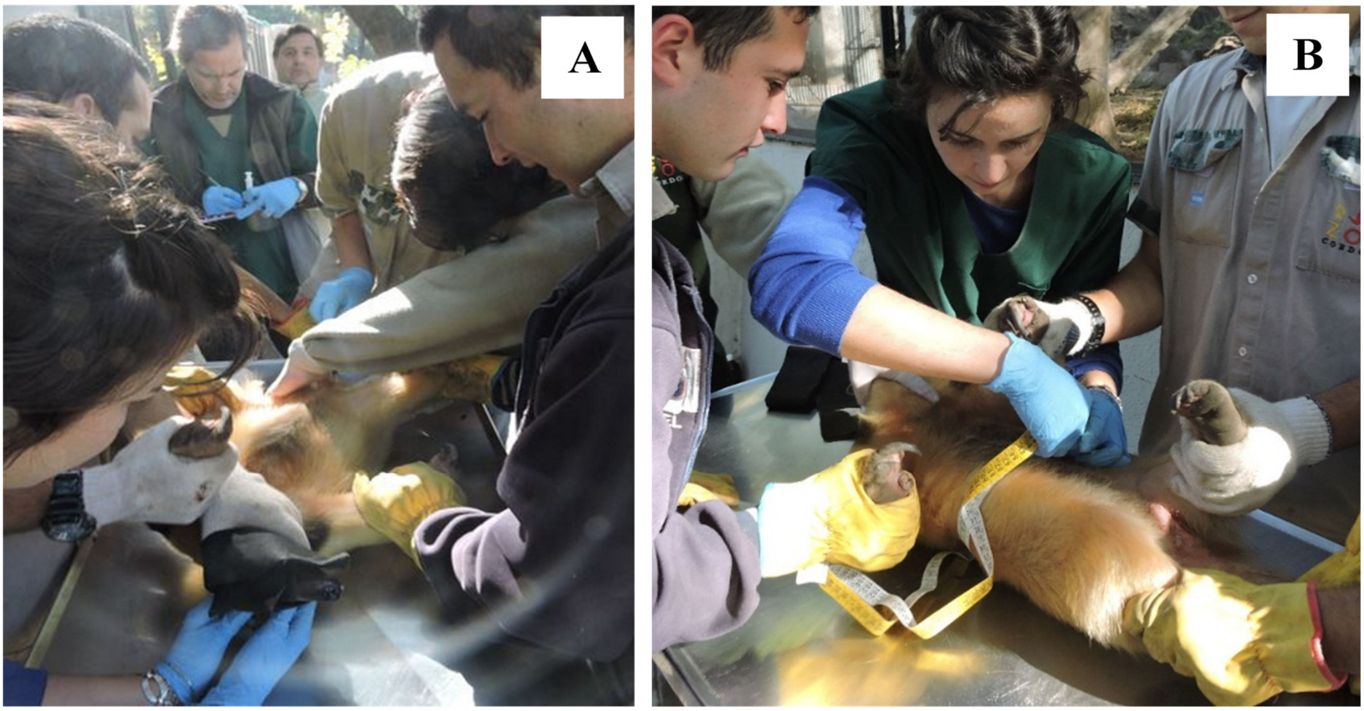

Figure S1. Veterinary check of adult *Tamandua tetradactyla* during winter (23 August 2016) of Study 2. Panel A shows the moment in which blood extraction from Male #3 is carried out and Panel B in which abdomen circumference from Male #2 is determined. Photos by Franco Rios.

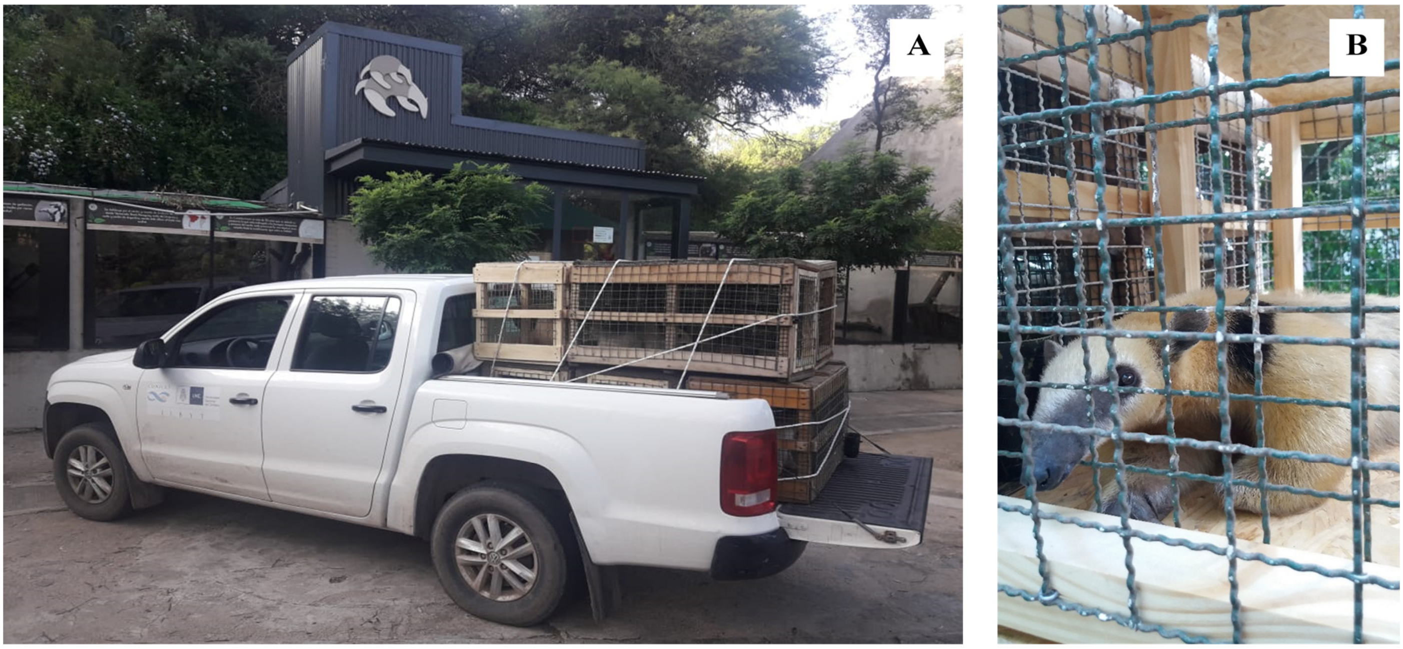

Figure S2. Transport of adult *Tamandua tetradactyla* during Study 3. Panel A shows how animals were transported ((Summer and Winter). Panel B shows Female #2 inside the box, previous to winter transport (29 July 2019). Photos by Gabina Eguizábal.
